# Modification of Muscle Proteins Induced by Novel Non-Thermal Processing: Theory, Characterization, and Consequences

**DOI:** 10.3390/foods15050963

**Published:** 2026-03-09

**Authors:** Yulong Bao, Hao Gou, Wanjun Xu, Longteng Zhang, Yuemei Zhang, Hui Hong, Yi-Ming Zhao

**Affiliations:** 1 School of Food and Biological Engineering, Jiangsu University, Zhenjiang 212013, China; gouhao@stmail.ujs.edu.cn; 2 Beijing Laboratory for Food Quality and Safety, College of Food Science and Nutritional Engineering, China Agricultural University, Beijing 100083, China; x1779961516@163.com (W.X.); hhong@cau.edu.cn (H.H.); 3 School of Food Science and Engineering, Hainan University, Haikou 570228, China; zhanglongteng@hainanu.edu.cn; 4 Key Laboratory of Geriatric Nutrition and Health (Beijing Technology and Business University), Ministry of Education, Beijing 100048, China; zhangyuemei@btbu.edu.cn

**Keywords:** non-thermal processing, protein oxidation, protein denaturation, protein charge, protein cross-linking

## Abstract

Muscle-protein modification plays a critical role in determining the quality, functional properties, and nutritional value of meat and aquatic products. Over recent decades, non-thermal processing technologies including irradiation, cold plasma, high-pressure processing, ultrasound, and electromagnetic fields have been widely explored in muscle foods. This review aims to critically examine modifications of food proteins subjected to non-thermal processing, with a focus on literature within the last five years. The review first introduces the type and theory of physicochemical modifications of food proteins, which includes protein oxidation, changes in net charge, cross-linking and aggregation. Subsequently, characterization methods applicable to food proteins are briefly discussed. Finally, the effects of non-thermal processing on muscle proteins are thoroughly discussed. This review will elucidate the intricate mechanisms of protein modification in muscle-based products, providing a theoretical framework to drive the advancement of innovative non-thermal processing technologies.

## 1. Introduction

Muscle foods, such as meat and fish, are highly perishable due to their rich nutrient content and high water activity, which create an ideal environment for microbial growth. Therefore, heat treatment is often required to eliminate harmful microorganisms. However, thermal processing can lead to the degradation of heat-sensitive nutrients such as vitamins and certain amino acids. Moreover, high-temperature processing may result in the formation of potentially hazardous compounds, including heterocyclic amines and polycyclic aromatic hydrocarbons. Excessive oxidation of lipids and proteins is another concern, which has been associated with deterioration in meat quality. In recent decades, non-thermal physical processing technologies have been used to improve the microbial safety and shelf life of muscle foods without relying on high temperatures [1]. In addition, non-thermal physical processing has also been used to improve other quality aspects of muscle foods, such as controlling ice crystals [2,3,4], facilitating marination [5,6,7], tenderizing tough meat [8,9], promoting the migration of active compounds from edible coatings to the food matrix [10], etc. Non-thermal processing is considered as a key innovation which may reshape meat industrialization in the intelligent era [11]. In muscle foods, the predominant component (other than water) is protein, and the presence of proteins is often accompanied by unsaturated lipids, reactive carbohydrates, catalytic ions, etc. These chemical compositions make muscle proteins prone to modification [12,13]. While non-thermal processing has been widely applied, its effect on muscle proteins received less attention compared to the effects of bacterial killing/inhibition. Therefore, there is a need to deepen the understanding of the mechanisms, characterization, and consequences of protein modification in muscle foods induced by novel non-thermal physical processing.

The field of non-thermal physical processing is rapidly evolving, and many excel-lent reviews [14,15,16,17,18] can be found in the literature about how food proteins are affected by non-thermal physical processing. With a continuous development of non-thermal technology and also advances in protein science, a better mechanistic understanding of protein modification due to non-thermal processing is needed. This review aims to summarize the mechanisms and factors governing the physics and chemistry behind food-protein modification. The consequences of the selected novel non-thermal processing technologies (ultrasound, electromagnetic field, high pressure, irradiation, cold plasma) on muscle proteins will also be covered in detail, with a focus on literature published in the last five years. The microbial inactivation or industrial implementation of non-thermal processing fall outside the scope of this review, and those aspects were covered in previous papers [19,20,21].

## 2. Theoretical Aspects of Protein Modification in Food System

**Figure 1:**
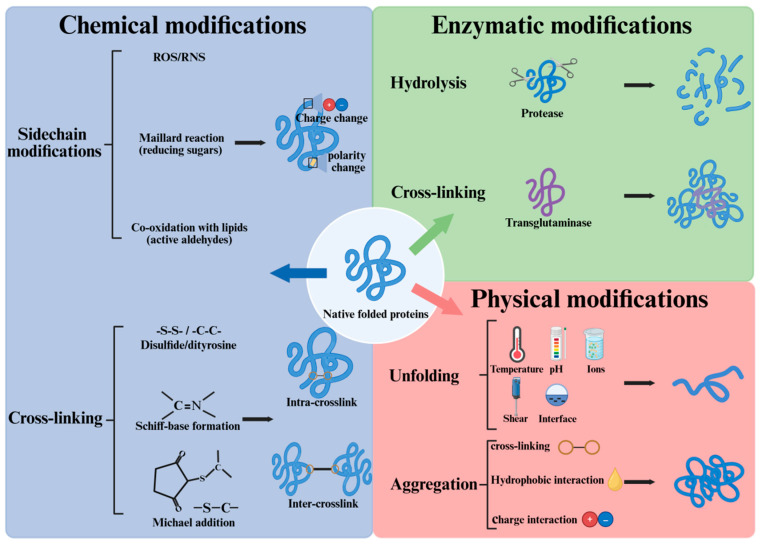
Illustration of common food-protein modifications during storage and processing.

Many native food proteins are marginally stable, and they are prone to various modifications during storage and processing (Figure 1). A theoretical understanding of protein modification will offer valuable insights into the effects of non-thermal processing on muscle proteins. Here we briefly summarize the main theory in the modification of food proteins.

### 2.1. Chemical Modifications

For proteins in vivo, various post-translational modifications (PTM) occur, and the PTMs can either activate or inhibit a protein’s function, impacting on its ability to interact with other molecules or perform its biological role. Common PTMs include phosphorylation, glycosylation, ubiquitination, acetylation, methylation, etc. [22]. A relatively new PTM, protein lactylation, was discovered on histones in 2019 [23], and Ma et al. [24] recently found that protein lactylation may participate in the meat-quality development in pork.

However, many of these active protein modifications are no longer possible or less likely in postmortem muscles when ATP has been used up. During storage and processing, oxidation and the Maillard reaction are two common chemical reactions involving food proteins [25]. Protein oxidation, by a broader definition, refers to all reactions which lead to the removal of electrons from protein. According to Estévez et al. [26], the Maillard reaction is closely linked to protein oxidation. For example, reactive di-carbonyls derived from the Maillard reaction can lead to oxidative deamination of food proteins, and the underlying chemical mechanism has been well discussed [27]. There is a coexistence of proteins, lipids, carbohydrates, catalytic ions, etc., in many foods, rendering the food proteins susceptible to oxidative stress. In normal viable cells, oxidative stress is well controlled by the dynamic cellular antioxidant system. In contrast, excessive oxidative stress can build up when the endogenous antioxidants in foods are exhausted. Our previous study showed that during the cold storage of pre-rigor fish muscle, a drastic decrease in intrinsic antioxidants occurred during rigor transformation, accompanied by mitochondrial damage. Moreover, food processing often aggravates protein oxidation, and this has been thoroughly reviewed in meat products [13].

Upon oxidative stress, essentially all amino acids can be oxidized [28]. Cysteine is prone to oxidation and determination of thiol groups is widely used to quantify protein oxidation in meat [29]. Another marker of protein oxidation is the formation of protein carbonyls, mainly derived from alkaline amino acids, especially lysine [26]. Bao et al. [27] addressed that protein carbonyl contains structurally and mechanistically different protein modifications. With extensive oxidation, food proteins can form various intra- and inter-molecular cross-linkages, including disulfide, dityrosine, Schiff-base, and Michael addition products. Excessive intermolecular cross-linking will reduce the protein solubility and contribute to protein aggregation. Other than covalent bonds, the formation of protein aggregates can be induced by hydrophobic interaction and electrostatic interaction. Chi et al. [30] summarized the mechanism and driving forces of protein aggregation in aqueous solution, and the authors pointed out that aggregation depends strongly on factors such as temperature, pH, salt concentration, salt type, surfactant, etc.

### 2.2. Physical Modifications

#### 2.2.1. Protein Denaturation

Denaturation can be described as a change in the native conformation of a (globular) protein due to a change in conditions. According to Sanfelice and Temussi [31], native proteins are just marginally stable as the thermodynamic stability of folded proteins is slightly higher than that of the unfolded ones, and subtle changes in the environment may be sufficient to induce protein denaturation. The most relevant causes for denaturation of food proteins include temperature, pH, solvent composition, interface, or mechanical stress [32].

It is well known that food proteins undergo thermal denaturation. Although many modern physical processing can be categorized as non-thermal technology, transition local high temperature can occur, such as in ultrasound [33]. Compared to thermal denaturation, protein cold denaturation is less well known. At decreased temperature, protein conformational entropy overtakes the stabilizing hydrophobic effect, resulting in protein unfolding [34].

Balny and Masson [35] pointed out that pressure-induced protein denaturation is a complex phenomenon depending on many factors, including protein structure, pressure, temperature, pH, presence of sugars, salts, etc. Harano et al. [36] indicated that high-pressure-induced protein denaturation is driven by an increase in water entropy in the whole system. High pressure leads to a moderately swollen protein structure with a much larger water-accessible surface area as compared to the native protein. Although the translational and rotational motions of water penetrating the protein interior or at the protein surface are largely restricted, translational restriction for water molecules that are sufficiently far from the protein is greatly reduced, therefore the entropy of water in the whole system increases upon pressure denaturation.

It is known that proteins can be denatured by adsorption on interfaces, especially hydrophobic ones [37]. Protein denaturation at interfaces has been extensively studied, and it has been reviewed with regard to different interfaces, including oil–water [38], ice-–water [39], air–water [40], etc. As summarized by Walstra and De Roos [34], protein molecules can adsorb onto the interface with different segments, and it is mainly the hydrophobic groups that are adsorbed, and therefore the proteins must change their conformation as most hydrophobic groups are buried in the interior of the native protein. The extent of conformational change depends on the stability of the protein itself, which is affected by temperature, pH, ionic strength, etc. For example, β-casein, an intrinsically disordered protein, unfolds to a considerable extent. Once adsorbed, protein unfolding may continue as the process takes time. The extent of unfolding depends on factors such as available surface area, protein concentration, etc. As the denaturation at the interface is generally irreversible, expansion and subsequent recompression of the interface may lead to significant protein denaturation. Other than these general mechanisms, interfacial areas may have a distinct microenvironment and lead to protein denaturation. One such example can be found in Arsiccio and Pisano [39], where a possible mechanism of ice-induced protein denaturation was discussed.

Gorelov and Morozov [41] used protein monocrystals to study the mechano-chemistry of protein; the ordered packing of molecules allows a uniform distribution of load. It was proposed that extension led to loss of physical contact between globules and stretching the covalent cross-links, and this caused protein denaturation. Proteins experience shear stress during processes such as mixing, centrifugation, pumping, etc. It was demonstrated that high shear force or high shear rate alone only led to slight conformational changes [42]. The same authors suggested that the presence of an air–liquid interface may play a role, and therefore they investigated the combined effects of shear and interface on the denaturation of recombinant human growth hormone (rhGH) and recombinant human deoxyribonuclease (rhDNase). The results showed that aggregation of rhGH only occurred in the presence of air–liquid interface, and the extent of aggregation increased at higher shear rate. In contrast, rhDNase, a less surface-active protein as compared to rhGH, did not aggregate even at high shear. When surfactant was used, aggregation of rhGH was significantly reduced, likely due to the competitive occupation of the inter-face by the surfactant. Jaspe and Hagen [43] tested whether a high shear can destabilize horse cytochrome c, a small globular protein. No evidence of protein denaturation was observed even at the highest shear rates. The authors suggested that very-high-molecular-weight protein such as multimeric proteins, or high solvent viscosity may result in protein denaturation under high shear.

**Figure 2:**
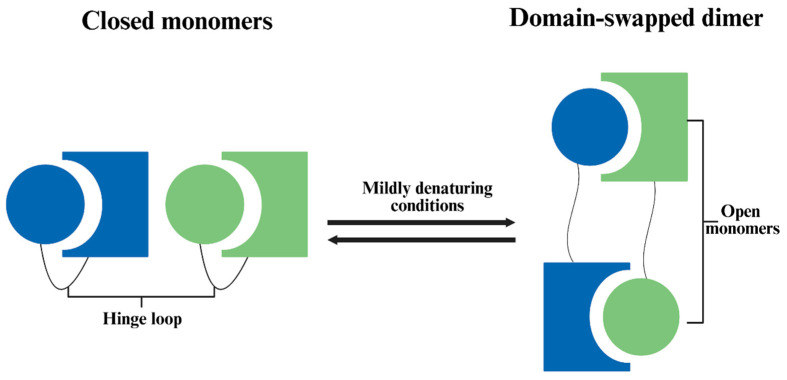
Illustration of 3D domain swapping of proteins. Proteins may partially unfold at mildly denaturing conditions, and when the denaturing conditions change, proteins may refold. The refolding process may form domain-swapped dimers or open monomers [44].

According to Bennett et al. [44], 3D domain swapping is one mechanism for protein oligomerization (Figure 2). In the process, the folded monomer is subjected to temporary conditions that favor partial unfolding. Then, when conditions are restored, one domain in the open monomer may be replaced by the same domain from another identical protein, and results in dimer or higher oligomer, especially at high protein concentration. The swapped “domain” can be a large tertiary globular domain or a small α-helix or β-sheet. One such example was given by Bennett et al. [45], where freezing–thawing led to dimerization of diphtheria toxin. It was suggested that freezing led to a pH drop of the phosphate buffer, which converted the protein into an open monomer, and the open monomers formed domain-swapped dimers when pH returned to normal during thawing.

#### 2.2.2. Changes in Protein Net Charge

Some of the amino acid sidechains can carry positive (His, Lys, Arg) or negative charges (Glu, Asp, Cys, Tyr), depending on their pKa values and the pH of the surroundings. Protein charge is largely determined by the amount of charged amino acid residues. The contributions from tightly bound metal, solvent, buffer and co-solvent ions are also significant [46]. According to Gitlin et al. [47], protein charge influences the structure, stability, activity and functionality of proteins. Protein charge can be affected by various chemical modifications induced by physical processing. The most frequent modification is protein oxidation, where a range of charged amino acid residues were affected. Protein carbonylation, one common indicator of protein oxidation, often led to a decrease in positively charged amino acids (Lys, Arg, His). This would alter the net charge, and Yu et al. [48] showed that oxidation can either increase the net negative charge (surrounding pH > pI) or decrease the net positive charge (surrounding pH < pI) of myofibrillar proteins. According to de Graff et al. [49], modifying just one charge of highly charged proteins with smaller radii of gyration can lead to a great change in protein stability. The relationship between protein oxidation and protein charge in muscle proteins has been thoroughly discussed by Bao et al. [50]. Dignon et al. [51] pointed out that in addition to net charges, the arrangement of charged amino acids (charge patterning) has an important role in determining the propensity of proteins to self-associate, aggregate, or phase separate.

## 3. Characterization of Common Food Protein Modification

### 3.1. Sidechain Modification

The most common assays for the determination of protein oxidation in meat have focused on thiol groups and carbonyl groups. A thiol (R-SH) is a carbon-bonded sulfhydryl group, and it is highly susceptible to oxidation. Loss of the thiol group is widely used as an indicator of protein oxidation. The predominant method for the detection of thiol groups is a spectrophotometric method based on DTNB [52]. Formation of protein carbonyls (C=O) is another general marker for protein oxidation in meat [53]. The traditional method for measuring protein carbonyl is based on DNPH derivatization. According to Estévez et al. [26], this method is unable to distinguish protein carbonyls which are formed by different pathways. Protein carbonylation can be induced by free radicals, lipid oxidation, and the Maillard reaction (glycoxidation). Both primary (mainly linked to the radical pathway) and secondary protein carbonyls (mainly linked to lipid oxidation pathway) can be formed in food proteins, and the Maillard-reaction-induced protein carbonylation has received less attention and is worth investigating. To gain specificity and information of a mechanistic nature, the analysis of specific protein carbonyls such as α-aminoadipic semialdehyde (AAS) and γ-glutamic semialdehyde (GGS) should be performed.

### 3.2. Protein Net Charge

Measuring the net charge of a protein is critical for understanding its solubility, stability, and functionality in various foods. One common method for assessing protein net charge is based on electrophoretic mobility, particularly through techniques such as zeta-potential analysis. In an electric field, proteins migrate depending on their net charge [54]. This method is sensitive to the ionic strength and pH of the buffer. Another classical approach involves titration-based methods, such as potentiometric titration or electrophoretic titration. These techniques determine how a protein uptakes or releases protons as the pH is gradually changed. Zahler and Shaw [46] pointed out that some of the earliest measurements using pH titrations ignored the tightly bound cations and anions, and there might be pH-dependent conformational changes (hence the changes in pKa of residues). As the protein net charge depends on the isoelectric point (pI), Isoelectric focusing gel electrophoresis, which separates proteins based on their pI in a pH gradient, is also widely used for evaluating net charge. And this technique can be coupled with Western blot or MS/MS to further identify the proteins [50].

Polyelectrolyte titration offers easy access to the determination of the surface charge of proteins and other biopolymers. In general, the protein solutions were incubated in excess with an oppositely charged polyelectrolyte, and the residual amount was back-titrated using o-toluidine blue for endpoint detection [55]. By incubating myofibrils with different dyes (positively charged Sarfarin O, neutral bromophenol blue, and negatively charged Orange G), Yu et al. [48] developed a method to estimate the net charges of myofibrils based on the amount of bound dyes. The net charge can also be estimated at the myofilament level based on the determination of Donnan potential [56]. The above-mentioned classical methods like electrophoresis and titration remain useful, while cutting-edge tools such as charge-detection MS [57] and computational predictions [58] are redefining the accuracy and efficiency of charge characterization.

### 3.3. Structural Characterization

Assessing protein secondary and advanced (tertiary and quaternary) structures is fundamental, as the techno-functionality of protein is tightly coupled to its structure. Various biophysical or spectroscopic methods including circular dichroism spectroscopy, Fourier-transform infrared spectroscopy, Raman, intrinsic fluorescence, NMR, X-ray crystallography, and cryo-electron microscopy, have been developed to characterize these structural features. A certain technique only works for a special protein system. Collagen has a special triple-helix structure, and therefore, optical rotation can be employed to characterize the structural changes during gelatin gelation [59].

**Figure 3:**
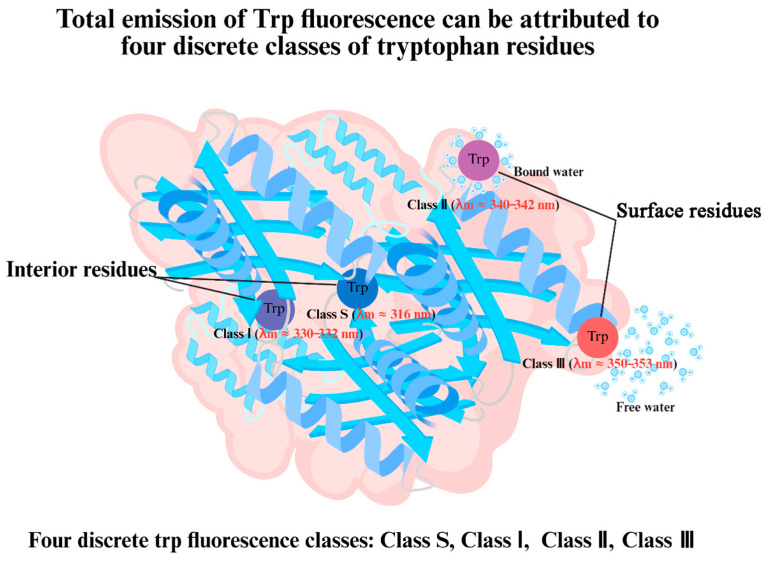
Interpretation of Trp fluorescence in terms of the micro-environment of Trp residues.

In the field of food science, these techniques can offer more mechanistic insights if experimental results are well interpreted. For example, in the analysis of intrinsic fluorescence, Reshetnyak and Burstein [60] suggested that the total emission of Trp fluorescence can be attributed to four discrete classes of tryptophan residues, designated S, I, II, and III. Classes S and I correspond to residues located in the interior of the protein, while classes II (contact bound water) and III (contact free water) correspond to the tryptophan residues located on the molecular surface (Figure 3). These authors suggested that an increased contribution from class S led to a blue shift in the fluorescence spectrum. Another example is in the determination of secondary structures using the Raman Spectrum of Proteins, where ranges other than the widely used 1600–1700 cm^−1^ can provide additional structural features, such as disulfide stretch around 500 cm^−1^ [61]. Zhu et al. [62] quantified the distribution of the disulfide bond conformations (gauche-gauche-gauche, trans-gauche-gauche, and trans-gauche-trans) and thereby obtained mechanistic insights about why garlic juice and allicin can stabilize meringues.

In recent years, computational approaches have dramatically advanced the field. The development of AlphaFold2 and RoseTTAFold has revolutionized tertiary structure prediction from sequence data. These AI-driven models have achieved near-experimental accuracy in many cases and are now widely used to complement experimental methods. One thing worth pointing out is that in food systems, proteins would seldom exist in the state of thermodynamic equilibrium, while this is often the presumption of many computational approaches.

### 3.4. Protein Cross-Links and Aggregation

Assessing protein cross-linking and aggregation is essential in studying protein functionality. Cross-linking involves the formation of covalent bonds between amino acid residues either within a single protein or between different protein molecules, while protein aggregation involves the association of protein molecules into larger assemblies through non-covalent or covalent interactions.

Cross-linking types in meat includes disulfide, dityrosine and carbonyl-involved cross-link. The detection of disulfides can be achieved through the following steps: blocking of free thiols, reduction in existing disulfide, and detection of newly formed thiols. In Raman spectroscopy, deconvolution of the signal between 490 and 550 cm^−1^ provides information about the relative abundance of each disulfide conformation; from most to least energetically stable: SSg-g-g (496–513 cm^−1^), SSt-g-g (514–527 cm^−1^), and SSt-g-t (527–548 cm^−1^) [62].

Intermolecular protein cross-linking leads to increased molecular weight and therefore, by comparing the SDS-PAGE profile of proteins in the presence or absence of a reducing agent, information about the cross-linking type can be obtained. Disulfide cross-linking can be both intra- and inter-molecular. Diagonal PAGE has been used as a tool to distinguish the two types of protein cross-linking [63]. Some muscle proteins have a relatively large molecular weight and protein cross-linking leads to larger and less soluble molecules, thereby making it difficult for the oxidized proteins to enter SDS-PAGE gel. A vertical agarose gel electrophoresis system was established by Warren et al. [64], providing the necessary resolution for the analysis of high-molecular-weight products. Another way to separate and analyze a large protein complex can be achieved with field-flow fractionation techniques [65].

## 4. Effects of Non-Thermal Processing on Muscle Proteins

Over the years, non-thermal processing technology has been widely applied in muscle foods; here we will focus on more recent studies about non-thermal processing, and emphasize their effects on muscle protein. For more complete background knowledge on how non-thermal processing affects protein functionality, readers are encouraged to refer to the following excellent reviews. (Ultrasound: [66,67]; electromagnetic field [68,69]; HPP: [70,71]; irradiation: [72,73]; cold plasma: [74,75], just to name a few examples).

### 4.1. Ultrasound

Ultrasound technology has gained increasing attention as a non-thermal method for modifying food proteins. It primarily uses high-frequency sound waves (typically 20 kHz–100 kHz) to induce physical and chemical changes in protein structures. Ultrasound generates acoustic cavitation and it can lead to localized high temperature and pressure, shear forces, and the generation of free radicals. As discussed earlier in Section 2, all these three aspects (high temperature/pressure, shear force, free radicals) can result in protein modification, being physical or chemical.

Ultrasound has been used in combination with other processes to enhance mass/heat transfer [76,77]. For example, ultrasound technology has been used to facilitate protein extraction [78], marination [79], freezing [80], thawing [81], etc. It has also been widely used as a homogenization technique to improve colloidal stability. Gul et al. [82] applied ultrasound and high pressure to improve the stability of sesame paste against phase separation, and the results showed that oxidative stability was also enhanced.

The intense physical conditions generated by ultrasound can lead to chemical modifications of amino acid sidechains, particularly those that are reactive or surface-exposed. Sidechain modification has been evidenced by decreased thiol groups [78,83,84] and increased carbonyl groups [85,86] in meat proteins. In some studies, an increase in thiol groups was reported [87,88], which may be due to the newly exposed thiol groups upon protein unfolding (Table 1). The protein unfolding process may also account for the increased surface hydrophobicity [89,90]. Zeta potential was found to be increased after ultrasound treatment [89]. Cavitation-induced shear and pressure disrupt non-covalent interactions such as hydrogen bonds, hydrophobic interactions, and van der Waals forces, and thereby significantly alter the advanced structure of food proteins. However, there is no general consensus in the literature about how ultrasound affects secondary structures of myofibrillar proteins [91,92,93,94]. Nevertheless, particle size was decreased, which may be beneficial for solubility and functionality [95,96]. However, excessive ultrasound intensity or prolonged treatment may lead to irreversible denaturation or loss of functionality [78,85,97].

During ultrasound processing, the enhanced mass/heat transfer and hence shorter processing time should result in limited protein modification, while the cavitation effect generally led to protein modification. Recent developments include the use of multifrequency ultrasound systems to fine tune the balance between the two opposing effects on protein modification. In the ultrasound-assisted freezing of shrimps, Xu et al. [80] combined different frequencies of ultrasound and achieved faster freezing and less ultra-structural damage of muscle tissue.

### 4.2. Electromagnetic Fields

Electric fields and magnetic fields fall under the umbrella of electromagnetism, and both can be applied in static, alternating, and pulsed fields. These technologies use electrical energy to achieve microbial inactivation and enzyme denaturation, thereby preserving the food color, flavor, and nutrients [100,101]. The electromagnetic field has been widely used in meat or meat products (Table 2). A large proportion of research has focused on the control of ice crystals, since the electromagnetic field can interact with the water dipole. For example, the magnetic field is believed to be able to break large water clusters into smaller ones and strengthen the hydrogen bonding between water and proteins (hydration shell). This is critical for protein stability.

On top of the dipole polarization of H_2_O, Sun et al. [111] argued that ions moving under an electric field is the main factor influencing the properties of NaCl solution during freezing. Therefore, redistribution of ions should be considered in dealing with protein functionality under electromagnetic fields. Other than ions and small polar molecules, electric fields can interact directly with the dipole moments of amino acid residues, forcing them to align with the field and therefore unfold the proteins.

Unlike electric fields, magnetic fields do not generate heat or violently rupture bonds. Instead, they influence the spatial arrangement of molecules. A strong magnetic field can force peptide chains to align parallel or perpendicular to the field, promoting ordered structures rather than random aggregation. Magnetic fields promote the unfolding of MP and sarcoplasmic proteins like myoglobin, indicated by a general increase in reactive sulfhydryls and surface hydrophobicity. Combining the magnetic field with additives like sodium tripolyphosphate (STPP) or calcium chloride can further optimize protein functionality. For example, AMF electromagnetic fields and STPP work together to enhance protein phosphorylation and solubility in low-salt systems. Wu et al. [108] suggested that magnetic fields could facilitate the rearrangement of the Mb structure, resulting in the transfer of its internal reactive groups to the external environment. This contributed to hydration and cross-linking between MP. Magnetic fields can influence the spin state of electrons, potentially slowing down oxidative reactions involving free radicals.

### 4.3. High-Pressure Processing (HPP)

High-pressure processing (HPP) uses very high pressure (often 300 to 800 MPa) for a short time to kill the microorganisms in foods and is best suited for liquid foods and highly perishable food products [112]. HPP fundamentally alters muscle proteins by disrupting the delicate balance of inter-molecular forces (hydrogen bonds, hydrophobic interactions, and ionic bonds) that hold them in their native structures. HPP can lead to the depolymerization of muscle filaments, releasing free myosin and G-actin [113]. With increasing pressure, the quaternary and tertiary structures begin to collapse, while at very high pressures, secondary structures such as the α-helix often transforms into random coils or β-sheets, which favors protein aggregation [114,115].

As reported by Zhang et al. [116], HPP-induced changes in myofibrillar protein conformation occur systematically: quaternary structures dissociate at 100–200 MPa, followed by significant tertiary disruptions above 200 MPa, and finally, secondary structural shifts at pressures between 300 and 700 MPa. Secondary structures are held by stable hydrogen bonds and hence they require higher applied pressure or holding time. These advanced structural changes lead to morphological changes at the microscopic level. For example, Xue et al. [117] showed that pressure greater than 200 MPa induced dimerization and swelling of myosin molecules extracted from rabbit muscle. Xue et al. [118] further suggested that different subfragments of myosin respond to HPP greater than 200 MPa differently; there were slight changes in light meromyosin as compared to heavy meromyosin.

Similar to myofibrillar proteins, sarcoplasmic proteins (globular proteins are highly sensitive to HPP) will denature with HPP. Myoglobin concentration decreased with increased HPP levels. Deoxymyoglobin and metmyoglobin content were greater at 600 MPa compared with non-pressurized control and other HPP levels [119]. This is the primary driver for changes in meat color and light scattering of HPP meat products. HPP can generate free radicals, especially at higher pressures (e.g., above 400–600 MPa) and temperatures, leading to oxidative stress, particularly in lipid-rich foods such as meat. By disrupting cell membranes and releasing catalytic iron, HPP initiates lipid and protein oxidation. Decreases in total thiols [114,120,121,122] and increases in disulfide bond [123] have been reported (Table 3).

### 4.4. Irradiation

In food processing, non-thermal irradiation technology generally includes gamma irradiation, electron-beam irradiation and UV irradiation. These technologies have been widely used to destroy bacteria, viruses, or insects that may be present in food. Irradiation may promote reactions within the food components due to the effect of the electron beam or free radicals from the radiolysis of water [129,130]. As can be seen in Table 4, irradiation may result in various physicochemical changes in muscle proteins, including denaturation, oxidation, aggregation, and fragmentation, etc. These physicochemical changes, at a mild level of irradiation, often led to improved protein functionality. Irradiation of myofibrillar proteins extracted from various sources has been shown to improve protein functionalities such as gelling, foaming, and emulsifying [131,132,133]. Both the protein and the irradiation source have an impact. As compared with myofibrillar proteins, the extracted myosin was more easily destroyed [134]. Compared with γ- ray irradiation, the electron-beam irradiation had a greater impact on the physicochemical properties of MPS and MS [134]. Patil and Khandekar [135] pointed out that LED technology will be an upcoming non-thermal food preservation method, which utilizes ROS to primarily target proteins and lipids.

Dąbrowska-Gralak et al. [137] found that the thermal stability of collagen type-I was not significantly affected upon irradiation at a dose range up to 50 kGy. However, oxidative modification was observed, evidenced by the introduction of new oxygen-containing functional groups in collagen. And the authors suggested that humidity played a key role regarding the amount and the rate of oxidative damage. Stanca et al. [138] compared the effects of gamma irradiation on the structure and stability of keratin, collagen, and gelatin. Results showed that irradiation led to an increase in random coil structures of keratin at the expense of β-sheet structures, accompanied by a decreased thermal stability. In contrast, no significant change in structure or stability was found for irradiated collagen and gelatin. The authors suggested that proteins with higher degree of ordered structures are more stable against gamma irradiation.

### 4.5. Cold Plasma

Plasma is composed of reactive species (ROS/RNS), charged particles, neutral atoms, etc., and it is commonly categorized into non-thermal (cold) and thermal types [74]. The former is particularly suitable for heat-sensitive materials due to its low-temperature operation. The generation of RNS is of particular interest to the red meat industry, as it can replace or reduce the use of nitrate/nitrite in meat products. ROS and RNS readily oxidize susceptible amino acids, particularly those with sulfhydryl (thiol) groups like cysteine and often leads to the conversion of sulfhydryl groups into disulfide bonds, promoting protein cross-linking [139]. At higher intensity of cold plasma treatments, severe protein oxidation can occur, which is often suggested by the formation of protein carbonyls. The decrease in thiol groups and the increase of carbonyl groups in muscle proteins following cold plasma treatments have been widely reported [75,140,141] (Table 5). Jiang et al. [142] also detected the formation of dityrosine in duck myofibrillar proteins. All these oxidative damages are associated with protein aggregation and loss of solubility, the latter being indicated by increased particle size [143]. Cold plasma treatment alters both the secondary and tertiary structures of meat proteins. Typically, a decrease in α-helix content and a concurrent increase in β-sheets and random coils were observed. This transition indicates an unfolding of the rigid protein structure, making it more flexible [144]. The protein unfolding is supported by the observation that surface hydrophobicity of myofibrillar proteins became larger after the treatments [145,146,147]. In contrast, surface hydrophobicity was found to be reduced after cold plasma treatment [148], possibly due to the hydrophobic interaction among unfolded myofibrillar proteins.

Both the sidechain modification and structural changes are vital for the techno-functionality of cold-plasma modified muscle proteins. The effect on solubility is dose-dependent; mild treatment can increase solubility by exposing hydrophilic groups and causing partial unfolding that facilitates water interaction [152], while severe treatment leads to excessive oxidation and extensive cross-linking, resulting in the formation of large, insoluble aggregates and reduced solubility. Cold plasma has shown promise in improving the gelling properties of meat proteins, particularly in surimi [153,154,155] and myofibrillar protein isolates [142,149].

A moderate “opening up” of the protein structure also allows a better adsorption at oil–water or air–water interfaces, which is beneficial for emulsification and foaming. Despite the potential in enhancing techno-functionality of muscle proteins, cold plasma may negatively impact sensory qualities such as the oxidation of myoglobin to metmyoglobin, leading to surface discoloration in red meats like beef [156].

## 5. Conclusions

The transition toward non-thermal physical processing represents a significant shift in meat and aquatic research. It is well known that non-thermal processing technologies, such as irradiation, ultra-sound, cold plasma, high-pressure processing (HPP), and electromagnetic fields, offer a pathway to enhance microbial safety and preserve the nutritional integrity of muscle foods. The less well known (or often ignored) aspect of non-thermal processing is that the molecular landscape of muscle proteins can undergo significant alterations, both chemically (oxidation, the Maillard reaction, etc.) and physically (denaturation, aggregation, etc.).

Depending on the operating parameters of non-thermal processing, excessive processing can lead to detrimental aggregation and loss of solubility; mild to moderate treatments often improve gelling, foaming, and emulsifying properties by inducing partial unfolding and exposing reactive functional groups. The phenomenon has been widely reported, yet the threshold for “excessive processing” is far from clear. By applying advanced characterization tools and AI-driven modeling tools, the underlying mechanism for muscle-protein functionality will be approached. A deeper theoretical understanding of how non-thermal energy interacts with muscle proteins (myofibrillar and sarcoplasmic proteins, extracellular proteins such as colla-gen and elastin) will empower the development of the next generation of meat and aquatic products.

## Figures and Tables

**Table 1:** Effects of ultrasound processing on the properties of muscle proteins.

Protein Source	Processing Parameters	Main Effects	Reference
MP from black soldier fly	300, 500, 700, 900 W 20 kHz; 20 min	↑: Carbonyl content, Surface hydrophobicity ↓: Total thiol group, Particle size, Turbidity, Intrinsic fluorescence intensity	[78]
MP from *Neosalanx taihuensis*	200, 400, 600 W, amplitude 60% 20 kHz; 60 min	↑: Intrinsic fluorescence intensity, β-sheet Surface hydrophobicity, Carbonyl content ↓: Total thiol group, α-helix	[83]
MP from tuna	160, 280, 400 W 40 kHz; 12 min	↑: Surface hydrophobicity ↓: Total thiol group, Particle size	[84]
MP from porcine ham muscle	200 W, amplitude 80% 20 kHz; 0–120 min	↑: Carbonyl content, Surface hydrophobicity α-helix, ↓: Turbidity, Particle size, Random coil, β-sheet	[85]
MP from bay scallop	150, 350, 550 W, amplitude 60% 20 kHz; 60 min	↑: Carbonyl content, Intrinsic fluorescence intensity, β-sheet, β-turn ↓: α-helix, Random coil	[86]
MP from shrimp *Litopenaeus vannamei*	400 W 20 kHz; 0–15 min	↑: Surface hydrophobicity, Free sulfhydryl content, Solubility, Zeta potential ↓: α-helix, Random coil, Particle size	[87]
MP from White croaker frozen surimi	500 W, intensity 30% 20 kHz; 2–10 min	↑: Total thiol group, Surface hydrophobicity	[88]
Protein from *Tenebrio molitor*	100, 200, 300, 400, 500 W; 20 min	↑: Free sulfhydryl content, Zeta potential Surface hydrophobicity, α-helix ↓: Particle size, Turbidity, Intrinsic fluorescence intensity, β-turn, β-sheet	[89]
MP from *Tenebrio molitor*	300, 500, 700, 900 W Ice bath; 30 min	↑: Carbonyl content, Surface hydrophobicity ↓: Total thiol group, Turbidity, Particle size, Intrinsic fluorescence intensity	[90]
Protein from *Solenaia oleivora*	200, 400, 600 W 20 kHz; 20 min	↑: Surface hydrophobicity, Solubility, Intrinsic fluorescence intensity, Zeta potential, β-turn, α-helix, ↓: Particle size, Total thiol group, Random coil	[91]
MP from *Tenebrio molitor*	500 W, amplitude 20% 20 kHz; 0–30 min	↑: Intrinsic fluorescence intensity, Solubility, Zeta potential, Surface hydrophobicity, α-helix, β-turn ↓: Particle size, β-sheet	[92]
MP from pork *longissimus dorsi*	150, 300, 450, 600 W 20 kHz; 15 min	↓: α-helix	[93]
MP from White croaker frozen surimi	500 W 20 kHz; 2–10 min	↑: Solubility, Zeta potential, Random coil, β-turn, β-sheet ↓: Particle size, Intrinsic fluorescence intensity, α-helix	[94]
MP from *Sipunculus nudus*	750 W, amplitude 0–100%; 10 min	↑: Solubility ↓: Particle size	[95]
MP from fish *Coregonus* *peled*	150, 200, 250 W 20 kHz; 0–12 min	↑: Carbonyl content, Surface hydrophobicity, Solubility ↓: Particle size, Total thiol group	[96]
MP from Silver carp	130, 260, 390, 520 W 40 kHz; 30 min	↑: Solubility, Zeta potential, β-turn, β-sheet, Surface hydrophobicity, Intrinsic fluorescence intensity ↓: Particle size, α-helix, Random coil	[97]
MP from White croaker frozen surimi	500 W, amplitude 60% 20 kHz; 2–30 min	↑: Free sulfhydryl content, Solubility, Surface hydrophobicity ↓: Particle size	[98]
MP from mussel	150, 300, 450, 600 W 20 kHz; 16 min	↑: Solubility, Zeta potential, β-turn, Surface hydrophobicity, Free sulfhydryl content ↓: Particle size, Turbidity, Intrinsic fluorescence intensity, β-sheet	[99]

Arrows (↑ and ↓) indicate an increase or decrease in the corresponding parameter.

**Table 2:** Effects of electromagnetic field processing on the properties of muscle proteins.

Technology	Protein Source	Processing Parameters	Main Effects	Reference
Pulsed electric field	MP from pork tenderloin	Field strength 2 kV/cm 110.6, 141.2, 173.6 Hz Duty factor 2.3%	↑: Surface hydrophobicity, Carbonyl content, Random coil, β-sheet ↓: Solubility, Total thiol group, Particle size, α-helix, Random coil	[102]
	MP from pork leg muscle	Field strength 2.1 kV/cm Pulse width 100 μs, frequency 5 Hz 0–30 min	↑: Intrinsic fluorescence intensity, Solubility, α-helix ↓: Random coil, Surface hydrophobicity, β-sheet, β-turn	[103]
	MP from PSE-like chicken breast muscle	Field strength 8, 18, 28 kV/cm 800 Hz Duty factor 47%	↓: Particle size	[104]
	MP from pork *Longissimus lumborum*	Field strength 2.5, 5, 7.5 kV/cm Pulse width 6 μs, frequency 500 Hz 30, 60, 90 s	↓: Total thiol group	[105]
Magnetic field	MP from pork loin	Field strength 3, 6, 9, 12 mT 3 h	↑: α-helix, Surface hydrophobicity ↓: Intrinsic fluorescence intensity, β-sheet	[106]
	MP from pork tenderloin	Field strength 0–10.4 mT 3 h	↑: Turbidity	[107]
	Myoglobin from horse skeletal muscle	Field strength 9 mT 1 h, 4 °C	↑: Intrinsic fluorescence intensity ↓: Total thiol group	[108]
	MP from silver carp	Field strength 3, 6, 9, 12 mT 3 h	↑: Solubility, Surface hydrophobicity	[109]
	MP from pork *longissimus dorsi*	Field strength 5 mT 3 h, 4 °C	↑: Solubility, Surface hydrophobicity, Total thiol group ↓: Turbidity, Intrinsic fluorescence intensity, Particle size	[110]

Arrows (↑ and ↓) indicate an increase or decrease in the corresponding parameter.

**Table 3:** Effects of high-pressure processing on the properties of muscle proteins.

Protein Source	Processing Parameters	Main Effects	Reference
MP from bighead carp	300 MPa for 20 min 30 MPa homogenization, twice	↑: Zeta potential, Surface hydrophobicity, β-sheet ↓: Total thiol group, Particle size, α-helix, Random coil	[114]
Protein from silkworm pupae	300, 400, 500, 600 MPa 10 min, 25 °C	↑: Turbidity, β-sheet, β-turn ↓: Intrinsic fluorescence intensity, α-helix, Free sulfhydryl content, Surface hydrophobicity	[115]
MP from shrimp Penaeus vannamei	138 MPa Microfluidizer 0–10 cycles	↑: Intrinsic fluorescence intensity, Random coil ↓: Particle size, Turbidity, α-helix, Total thiol group, Disulfide-bond content	[120]
MP from fish Tai Lake whitebait	100, 200, 300, 400 MPa 10 min, 25 °C	↑: Carbonyl content, Surface hydrophobicity β-sheet, ↓: Total thiol group, Intrinsic fluorescence intensity, Random coil, α-helix, β-turn	[121]
MP from shrimp Litopenaeus vannamei	150, 300, 450, 600 MPa 10 min, 25 °C	↑: Carbonyl content, Surface hydrophobicity, β-sheet ↓: α-helix, Intrinsic fluorescence intensity, Total thiol group	[122]
MP from mud carp	100, 200, 300 MPa 3, 9, 15 min	↑: Surface hydrophobicity, Particle size, Disulfide bond content ↓: Turbidity, TCA-soluble peptide content	[123]
MP from pork ham	30, 60, 90, 120, 150 MPa Microfluidizer 3 cycles	↑: Surface hydrophobicity ↓: Particle size, Turbidity	[124]
MP from chicken breast	30, 60, 90, 120 MPa Microfluidizer 3 cycles	↑: Solubility, β-sheet, Surface hydrophobicity, ↓: α-helix, β-turn, Turbidity	[125]
Myoglobin from equine skeletal muscle	100, 200, 300, 400 MPa 20 min, 25 °C	↓: Intrinsic fluorescence intensity	[126]
Protein from oyster	20, 60, 100 MPa 3 cycles	↑: Surface hydrophobicity, Solubility, Random coil ↓: Intrinsic fluorescence intensity, α-helix	[127]
Mantle proteins from scallops	100, 200, 300, 400, 500 MPa 10 min, 6 °C	↑: Total thiol group ↓: Solubility, α-helix	[128]

Arrows (↑ and ↓) indicate an increase or decrease in the corresponding parameter.

**Table 4:** Effects of irradiation processing on the properties of muscle proteins.

Protein Source	Processing Parameters	Main Effects	Reference
MP from chicken breast	3, 5, 7, 15 kGy Dose rate 0.5 kGy/s	↑: Intrinsic fluorescence intensity, Surface hydrophobicity ↓: Turbidity, Particle size, Total thiol group	[131]
Protein from larvae *Protaetia brevitarsis*	5, 10 kGy Dose rate 5 kGy/h	↑: Solubility, Surface hydrophobicity ↓: Intrinsic fluorescence intensity	[132]
MP from pork ham	2.5, 5, 10 kGy Dose rate 5 kGy/h for gamma irradiation 3 kGy/h for X-ray irradiation	↑: α-helix ↓: Solubility, Particle size, Intrinsic fluorescence intensity, β-sheet	[133]
MP and myosin from grass carp	4, 6, 8, 10 kGy	↑: Total thiol group ↓: Particle size, Solubility, Ca^2+^-ATPase Activity	[134]
Collagen from tilapia skin	1, 3, 6, 9, 12 kGy Dose rate 1.2 kGy/h	↑: Carbonyl content, Surface hydrophobicity ↓: Free sulfhydryl content, Solubility	[136]

Arrows (↑ and ↓) indicate an increase or decrease in the corresponding parameter.

**Table 5:** Effects of cold-plasma processing on the properties of muscle proteins.

Protein Source	Processing Parameters	Main Effects	Reference
MP from red shrimp	35 kV 1–5 min	↑: Carbonyl content, Surface hydrophobicity, Turbidity, β-sheet, Random coil ↓: Ca^2+^-ATPase Activity, Total thiol group, α-helix, β-turn	[140]
MP from duck breast	0–40 s	↑: Carbonyl content ↓: Total thiol group	[141]
MP from duck breast	50 V 3 min	↑: Carbonyl content, Dityrosine content ↓: Total thiol group, Ca^2+^-ATPase Activity	[142]
Protein from chicken breast	750 W, 0–40 s	↑: Particle size, Surface hydrophobicity, Zeta-potential, Turbidity, α-helix ↓: Intrinsic fluorescence intensity, Solubility Total thiol group, β-sheet, Random coil	[143]
Tropomyosin from shrimp	50 kV 20 min	↑: Surface hydrophobicity, β-sheet, Random coil β-turn ↓: Intrinsic fluorescence intensity, α-helix	[145]
MP from hairtail fish	50 kV 30–300 s	↑: Surface hydrophobicity, Turbidity ↓: Total thiol group	[146]
MP from mandarin fish	650 W 0–12 s	↑: Turbidity, Surface hydrophobicity, β-sheet ↓: Intrinsic fluorescence intensity, Solubility, Total thiol group, β-turn, Random coil	[147]
MP from Atlantic salmon	320 W 5 min	↑: Intrinsic fluorescence intensity, Solubility, Dityrosine content, Carbonyl content ↓: Particle size, Surface hydrophobicity, Total thiol group	[148]
MP from chicken breast	Plasma jet: 40 V, 1.8 A 0–16 min	↑: Intrinsic fluorescence intensity, Surface hydrophobicity ↓: β-sheet	[149]
MP from pork *Lumbar longissimus*	50, 60, 70 kV 5 min	↑: Surface hydrophobicity, Carbonyl content ↓: Total thiol group, Particle size	[150]
MP from Asian Sea Bass	80 kV 5–15 min	↑: Carbonyl content ↓: Total thiol group	[151]

Arrows (↑ and ↓) indicate an increase or decrease in the corresponding parameter.

## Data Availability

No new data were created or analyzed in this study. Data sharing is not applicable to this article.
